# Successful Treatment and Retreatment With Erdafitinib for a Patient With *FGFR3-TACC3* Fusion Squamous NSCLC: A Case Report

**DOI:** 10.1016/j.jtocrr.2023.100511

**Published:** 2023-03-30

**Authors:** Chi Pham, Daenielle Lang, Wade T. Iams

**Affiliations:** Division of Hematology/Oncology, Department of Medicine, Vanderbilt-Ingram Cancer Center, Nashville, Tennessee

**Keywords:** FGFR3-TACC3 fusion, Non–small cell lung cancer, Targeted therapy, Case report

## Abstract

*FGFR3-TACC3* fusions have been identified in patients with multiple cancer types, and tumors with these alterations are potentially sensitive to selective FGFR inhibitors. However, there are no FGFR inhibitors approved by the U.S. Food and Drug Administration for the treatment of patients with NSCLC with *FGFR* alterations. Here, we report a case of a patient with *FGFR3-TACC3* fusion squamous NSCLC who achieved a radiographic response and disease control for 11 months on initial treatment with erdafitinib and subsequently obtained an additional 8 months of disease control after erdafitinib retreatment after 5 months of intervening chemotherapy. Further investigation into FGFR inhibitor treatment specifically and targeted therapy retreatment for patients with NSCLC may increase our therapeutic options for these patients.

## Introduction

Among the recently identified potentially therapeutically actionable pathogenic variants, *FGFR* alterations have been implicated in various cancer types (roughly 5%–10% of all cancers).^1^

Though not as common as *FGFR* amplification (66%) or single nucleotide variants (26%), *FGFR* fusions account for roughly 8% of all *FGFR* alterations, and *FGFR3-TACC3* fusions have been observed to be potentially associated with squamous NSCLC tumorigenesis.^1^^,^^2^ *FGFR* fusions are potentially therapeutically actionable, though there are no agents approved by the U.S. Food and Drug Administration (FDA) to target *FGFR* in patients with NSCLC. However, multiple selective FGFR–tyrosine kinase inhibitors (TKIs) are FDA-approved for other oncologic indications (erdafitinib, pemigatinib, infigratinib, and futibatinib).^1^ Previous studies have reported several cases of patients with NSCLC having *FGFR* alterations who achieved disease response on FGFR inhibitor treatment (pemigatinib and erdafitinib specifically) (Table 1).^3^, ^4^, ^5^, ^6^ Furthermore, there is a growing body of literature suggesting the potential therapeutic efficacy of targeted agent rechallenge (predominantly with the EGFR TKI osimertinib) after intervening therapy in patients with NSCLC.^7^^,^^8^

**Table 1:** Previous Studies Evaluating Disease Response to Treatment With FGFR Inhibitors in Patients With NSCLC Having FGFR Alterations

Study	Study Design	FGFR Status	Treatment	Disease Response
FIGHT-101 trial^3^	Phase 1/2 clinical trial	*FGFR3-TACC3* fusion	Pemigatinib	Patient had PD after 3 mo of treatment
FIGHT-101 trial^3^	Phase 1/2 clinical trial	*FGFR3 R248C* mutation	Pemigatinib	Patient had no measurable response
FIGHT-101 trial^3^	Phase 1/2 clinical trial	*FGF10* amplification	Pemigatinib	Patient had PD after 2 mo of treatment
Detection of known and novel FGFR fusions in non-small cell lung cancer by comprehensive genomic profiling^4^	Case report	*FGFR2-L2TFL1* fusion	Erdafitinib	Patient achieved PR after 2 mo of treatment and continued treatment for 11 mo
Remarkable response to erdafitinib in metastatic lung adenocarcinoma with FGFR fusion^8^	Case report	*FGFR2-BICC1*fusion	Erdaftinib	Patient achieved PR after 3 mo of treatment

PD, progressive disease; PR, partial response.

Here, we present a case of a patient with *FGFR3-TACC3* fusion squamous NSCLC who experienced radiographic response and disease control with erdafitinib treatment on two separate occasions. To our knowledge, this is the first report of successful erdafitinib treatment and retreatment for NSCLC with an *FGFR3-TACC3* fusion.

## Case Presentation

A 51-year-old woman with a 28 pack-years history of smoking presented to the emergency department for evaluation of upper back pain. Computed tomography imaging revealed a large left lower lobe lung mass and multiple bilateral pulmonary nodules. Biopsy of the left lung mass revealed squamous cell carcinoma that was p40-positive, consistent with NSCLC. Her initial TNM stage was IV (cT2bN0M1a). Broad-based next-generation sequencing (NGS) was not performed at this time.

**Figure 1:**
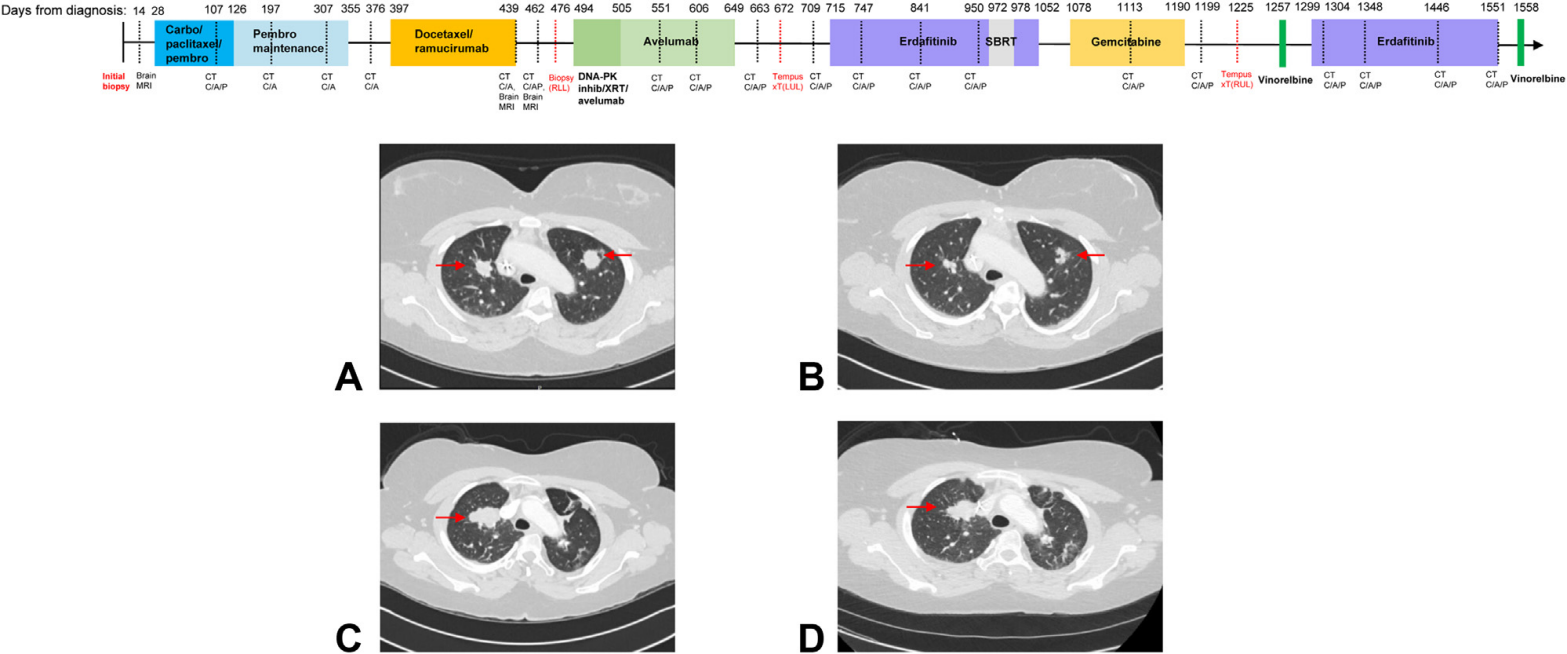
Treatment timeline and radiographic images on erdafitinib. (*A*) Pretreatment CT of the chest before first treatment with erdafitinib, which reveals bilateral upper lobe nodules. (*B*) Chest CT after 6 months of treatment, which reveals significant decrease in size of bilateral nodules. (*C*) Chest CT before rechallenge with erdaftinib (*D*) Chest CT illustrating stability after 5 months of retreatment with erdafitinib. Carbo, carboplatin; CT, computed tomography; Inhib, inhibitor; MRI, magnetic resonance imaging; Pembro, pembrolizumab; SBRT, stereotactic body radiotherapy; XRT, radiation therapy.

The patient received first-line chemoimmunotherapy with a partial response and subsequent disease control for 12 months. At first progression, she was treated with docetaxel plus ramucirumab with a radiographic response but developed substantial toxicities, which prompted a transition to a clinical trial with a novel agent plus avelumab and radiation. Her clinical trial treatment provided an additional 5 months of disease control. Figure 1 illustrates the full treatment timeline.

At the time of progression on the third line clinical trial, biopsy was performed on the patient’s left upper lobe tumor and sent for NGS testing with a 648 gene Tempus xT panel. Pathological findings revealed squamous NSCLC with an *FGFR3-TACC3* fusion and an *NFE2L2* R34Q mutation at 70.7% variant allele frequency with a tumor mutational burden of 4.7 mutations per megabase. Her case was reviewed by the institutional molecular tumor board, and FGFR inhibitor treatment was recommended. She began treatment with erdafitinib (8 mg daily) with a radiographic response on the first imaging and 11 months of disease control before further progression of her pulmonary masses (Fig. 1*A* and *B*). While on erdafitinib, the patient experienced fatigue, intermittent diarrhea, weight loss, hyperphosphatemia, corneal ulceration, onycholysis, and hand-foot syndrome during the first 3 months, which prompted a dose reduction to 6 mg for 1 month, followed by further dose reduction to 4 mg for 7 months. All the adverse events were grade 1 or 2, except her corneal ulceration (grade 3).

Subsequently, the patient received gemcitabine with 4 months of disease control, followed by 1 month of vinorelbine. At this time, a right upper lobe lung mass biopsy was pursued with broad-based NGS testing with the Tempus xT panel. Results revealed squamous NSCLC with an *FGFR3-TACC3* fusion and an *NFE2L2* R34Q mutation at 85.1% variant allele frequency with a tumor mutational burden of 6.3 mutations per megabase. Because of the previous prolonged disease control with erdafitinib and the patient's preference to retry oral targeted therapy, her case was again reviewed in the molecular tumor board. FGFR inhibitor rechallenge was noted to be a reasonable next line of therapy.

With an off-erdafitinib interval of 5 months, the patient restarted erdafitinib (8 mg daily, reduced to 6 mg daily 1 month after because of hand-foot syndrome) and experienced disease control (stable disease on radiographic imaging) for a total of 8 months (Fig. 1*C* and *D*).

## Discussion

Since their identification in 2012 in a patient with glioblastoma, *FGFR3-TACC3* fusions have garnered attention because of their sensitivity to selective FGFR inhibitors despite their low frequency recorded across human cancer types.^1^ In the NSCLC cohort, *FGFR3-TACC3* fusions have been recorded in 0.2% to 1.1% of screened populations, with the most common pathological subtype being squamous cell carcinoma.^2^^,^^4^ In addition, the emergence of *FGFR3-TACC3* fusions has been documented as an acquired resistance mutation during EGFR TKI therapy*.* In this context, successful combination treatment with an FGFR inhibitor and EGFR TKI has been observed.^9^ Here, we report the case of a patient with *FGFR3-TACC3* fusion squamous NSCLC who was treated with erdafitinib on two separate occasions, with an intervening period of chemotherapy, and achieved disease control for 11 months and 8 months, respectively, highlighting the potential clinical use of FGFR inhibitors in patients with NSCLC.

Erdafitinib is currently FDA-approved for patients with *FGFR2/3* alterations and locally advanced or metastatic urothelial carcinoma.^1^ In the era of widespread, broad molecular testing, we encounter rare mutations that are potentially actionable but not thoroughly evaluated in the specific histologic context in which they are identified. Furthermore, patients with certain targetable oncogenic mutations may have inferior results when treated with immunotherapy, limiting therapeutic options to targeted agents and chemotherapy.^10^ The emerging body of literature supporting both off-label targeted therapy use and targeted therapy rechallenge is of utmost importance to improve outcomes in these patients.

This case presents evidence for consideration of both treatment and retreatment with FGFR inhibitors in patients with *FGFR* fusion-driven NSCLC, specifically *FGFR3-TACC3*.

In conclusion, we present a case of a patient with squamous NSCLC with an *FGFR3-TACC3* fusion who achieved an initial response on erdafitinib treatment and further disease control on erdafitinib rechallenge. This provides evidence for off-label FGFR inhibitor use in settings in which a potentially actionable *FGFR* mutation is identified, and it supports a growing body of literature suggesting the potential utility of rechallenge with oral targeted therapies.

## CRediT Authorship Contribution Statement

**Chi Pham:** Conceptualization, Writing - original draft, Visualization, Project administration.

**Daenielle Lang:** Writing – review & editing, Visualization.

**Wade T. Iams:** Conceptualization, Resources, Writing – original draft, Writing - review & editing, Supervision.

## References

[1] Ellinghaus P., Neureiter D., Nogai H., Stintzing S., Ocker M. (2022). Patient selection approaches in FGFR inhibitor trials—many paths to the same end?. Cells.

[2] Wang R., Wang L., Li Y. (2014). FGFR1/3 tyrosine kinase fusions define a unique molecular subtype of non–small cell lung cancer. Clin Cancer Res.

[3] Subbiah V., Iannotti N.O., Gutierrez M. (2022). Fight-101, a first-in-human study of potent and selective FGFR 1–3 inhibitor pemigatinib in pan-cancer patients with FGF/FGFR alterations and advanced malignancies. Ann Oncol.

[4] Qin A., Johnson A., Ross J.S. (2019). Detection of known and novel FGFR fusions in non–small cell lung cancer by comprehensive genomic profiling. J Thorac Oncol.

[5] Urrutia Argueta S.A., Hanna N.H. (2020). Remarkable response to Erdafitinib in metastatic lung adenocarcinoma with FGFR fusion. JCO Precis Oncol.

[6] Loriot Y., Schuler M.H., Iyer G. (2022). Tumor Agnostic Efficacy and Safety of ERDAFITINIB in Patients with advanced solid tumors with prespecified fibroblast growth factor receptor alterations (FGFRalt) in RAGNAR: interim analysis results. J Clin Oncol.

[7] Fuchs V., Kian W., Lichtenberg R. (2022). Next-generation sequencing liquid biopsy-guided osimertinib rechallenge in EGFR-mutated advanced non-small-cell lung cancer patients. Clin Drug Investig.

[8] Metro G., Bonaiti A., Birocchi I. (2022). Tracking and tackling the Tumor Dynamics Clonal Evolution: osimertinib Rechallenge after interval therapy might be an effective treatment approach in epidermal growth factor receptor (egfr)-mutant non-small cell lung cancer (NSCLC). J Thorac Dis.

[9] Haura E.B., Hicks J.K., Boyle T.A. (2020). Erdafitinib overcomes FGFR3-TACC3–mediated resistance to osimertinib. J Thorac Oncol.

[10] Calles A., Riess J.W., Brahmer J.R. (2020). Checkpoint blockade in lung cancer with driver mutation: choose the road wisely. Am Soc Clin Oncol Educ Book.

